# Viral etiology and seasonality of influenza-like illness in Gabon, March 2010 to June 2011

**DOI:** 10.1186/1471-2334-14-373

**Published:** 2014-07-07

**Authors:** Sonia Etenna Lekana-Douki, Dieudonné Nkoghe, Christian Drosten, Edgar Brice Ngoungou, Jan Felix Drexler, Eric M Leroy

**Affiliations:** 1 Centre International de Recherches Médicales de Franceville, BP 769 Franceville, Gabon; 2 Ministère de la Santé Publique, BP 5978 Libreville, Gabon; 3 Institute of Virology, Bonn Medical Centre, Bonn, Germany; 4 Département Epidémiologie-Biostatistiques, Université de Medecine, Libreville, Gabon; 5 UMR (IRD 224 /CNRS 5290/UM1-UM2), Institut de Recherche pour le Développement, Montpellier, France

**Keywords:** Gabon, Surveillance network, Influenza-like illness, Viruses, Seasonality

## Abstract

**Background:**

Surveillance of influenza-like illness (ILI) in Central Africa began only recently, and few data are therefore available on the circulation of influenza virus and other respiratory viruses. In Gabon, a Central African country, we established a surveillance network in four major towns in order to analyze cases of ILI among patients who visited health centers between March 2010 and June 2011, and to determine the viral etiology.

**Methods:**

Nasal swabs were sent for analysis to the Centre International de Recherches Médicales de Franceville, where they were screened for 17 respiratory viruses in a multiplex real-time reverse transcription polymerase chain reaction for all pathogens according the following pairs: adenovirus/parainfluenza virus 4, respiratory syncytial virus/human metapneumovirus, parainfluenza virus 1/parainfluenza virus 2, pandemic influenza virus A/seasonal influenza virus A (H1N1, H3N2)/seasonal influenza virus B, human coronaviruses 229E/OC43, human coronaviruses NL63/HKU1, rhinovirus/human parechovirus, and enterovirus/parainfluenza virus 3.

**Results:**

We analyzed a total of 1041 specimens, of which 639 (61%) were positive for at least one virus. Three-quarters of the patients were children under five years old. We therefore focused on this age group, in which 68.1% of patients were positive for at least one virus. The most common viruses were adenoviruses (17.5%), followed by parainfluenza viruses (PIVs) 1–4 (16.8%), enteroviruses (EV) (14.7%), respiratory syncytial virus (RSV) (13.5%), and influenza virus (11.9%). The prevalence of some viruses was subject to geographic and seasonal variations. One-third of positive samples contained more than one virus.

**Conclusions:**

Like most studies in the world, the virus PIVs, EV, RSV, Influenza virus, HRV were predominant among children under five years old in Gabon. An exception is made for adenoviruses which have a high prevalence in our study. However adenoviruses can be detected in asymptomatic persons. These finding gave a better knowledge of the circulation and the seasonality of the viruses involved in ILI in Gabon.

## Background

Acute respiratory tract infections are a major cause of morbidity and mortality worldwide [1]. Most such infections are due to viruses [2], which can provoke epidemics and, in some cases, pandemics. For example, in November 2002 a novel coronavirus emerged in southern China, then spread rapidly throughout world in 2003, affecting 25 countries across the five continents. This coronavirus, SARS-CoV (Severe Acute Respiratory Syndrome Coronavirus), affected 8000 people, of whom almost 800 died [3]. Likewise, a highly pathogenic avian influenza virus A (H5N1) spread extensively in migratory birds and poultry across 64 countries in Asia, the Middle East, Europe and Africa [4]. The World Health Organization (WHO) reported 630 confirmed human cases of H5N1 infection, with a case fatality rate of approximately 60% and significant economic losses [4-6]. The pH1N1 pandemic that started in 2009 lasted 14 months, from June 2009 to August 2010, and was responsible for thousands of cases and deaths [7]. In August 2010, 214 countries and territories throughout the world reported 18 449 deaths from A(H1N1)pdm09 infection [8].

Cases of influenza-like illness (ILI), defined by WHO as fever (≥38°C) and cough, or sore throat, runny nose and headache, are regularly reported across the five continents. In 2008, an estimated 28 000 to 111 500 deaths among children aged less than 5 years were attributed to influenza-associated acute lower respiratory tract infections (ALRI), 99% of these deaths occurring in developing countries [9]. A meta-analysis included 51 studies of children hospitalized between 0 and 4 years of age with severe ALRI from 1995 to 2011 on the American continent (Canada, USA, Colombia, Brazil, Chile, Argentina), Europe (Spain, Germany, Austria, Switzerland, Greece), Africa (South Africa, Mozambique, Kenya), Asia (Israel, Jordan, India, China, South Korea, Japan, Burma, Malaysia) and Oceania (Indonesia, Australia). The main viral etiologies were adenovirus (8.8%), influenza virus (7%), and PIV (5.8%) [10]. In the Western Pacific region, the proportions of cases of ILI attributed to influenza virus between 2006 and 2010 were 57% in China, 19% in Japan, 7% in the Republic of Korea, 4% in the Philippines and Singapore, and 3% in Australia [11]. ILI and influenza virus infections usually peak during the winter months in temperate countries. In tropical countries, the most common viral etiologies of ILI reported in children under five years old are RSV, PIV-3, adenovirus and influenza virus, as in temperate regions [12]. Influenza virus accounts for 5% to 30% of cases of ILI worldwide [13-18].

Respiratory viruses are most active during the rainy seasons in tropical countries [12]. This is the notably case of influenza virus in Singapore and Senegal [19,20], influenza virus A and B in Brazil [21], influenza outbreaks in Nigeria and India [12], and RSV in South America and Asia [12]. Adenovirus appears to be endemic in Taiwan, Hong-Kong and India, while in Kenya its prevalence increases in the hot period from November to February [12].

Data on ILI and influenza virus circulation in Africa are inadequate. A study conducted in 15 African countries (Angola, Ivory Coast, Democratic Republic of Congo (DRC), Egypt, Ethiopia, Ghana, Kenya, Madagascar, Morocco, Nigeria, Rwanda, South Africa, Tanzania, Uganda, Zambia) between 2006 and 2010 showed that influenza viruses were responsible for 21.7% of cases of ILI, ranging from 6.7% in Angola to 40.4% in Madagascar. In this study, 48% of ILI patients were children under five years old [22]. A study conducted in Cameroon (central Africa) detected viral infections in 65.1% of ILI patients [23].

In Gabon, a tropical Central African country, we have previously reported the circulation of influenza virus A(H1N1)pdm09 [24]. Before, the circulation of influenza viruses had been little studied. In Gabon, the circulation of respiratory viruses was not a major public health interest because of the other infectious diseases that cause a febrile illness such as malaria. In a pandemic context, it proved important to know the circulation and prevalence of influenza viruses and others respiratory viruses. To determine the prevalence, etiology and seasonality of viral respiratory tract infections we set up an ILI surveillance system at four sites in Gabon from March 2010 to June 2011.

## Methods

### Study design

Gabon is a typical rain forest country of Central Africa, with 1 517 685 inhabitants and a surface area of 270 000 square kilometers. We set up a surveillance system for ILI in the capital Libreville and in three other towns in different parts of the country (Franceville, Oyem and Koulamoutou). Libreville has a population of about 538 195 inhabitants. The populations of the other three towns are as follows: Franceville (South East, semi-rural): 103 840 inhabitants; Oyem (North, rural): 35 241 inhabitants; Koulamoutou (South, rural): 16 270 inhabitants [25]. The study took place in three healthcare centers in Libreville and in the regional hospitals of the other three towns.

The Gabonese climate is equatorial: moist and hot, with an annual average rainfall of 1836 mm, an average temperature of 24.5°C, and an average relative humidity of about 70%. The year begins with a short dry season between January and February, followed by a long rainy season between March and May; a long dry season follows between June and September, then a short rainy season from October to December. The study was conducted from March 2010 to June 2011.

### Patients and specimens

Patients were enrolled in this study if they visited a participating health center for influenza-like illness, comprising fever (≥38°C) and runny nose, or fever and cough, or fever and sore throat. Nasal samples were collected systematically. The patient’s name, age, sex and travel history during the month before onset were recorded. Nasal swabs were placed in dry tubes and stored at 4°C until being sent to CIRMF, once a week, for analysis.

### Laboratory analysis

The swabs were placed in saline and nucleic acids were extracted with the EZ1 virus minikit V2.0 on an EZ1 Advanced Instruments (Qiagen) or with the QIAamp kit. After the emergence of pandemic influenza, diagnostic testing was based on real-time reverse transcription PCR (RT-PCR) using a one-step multiplex real-time RT-PCR kit (Qiagen) [26]. Each 25-μl reaction mixture contained 5 μl of eluted RNA or DNA and 5 μl of 5× One Step RT-PCR buffer containing 12.5 mM magnesium chloride, 400 μM each deoxynucleoside triphosphate, 40 ng of bovine serum albumin per μl, 0.4 μM primers and 0.2 μM probes, and 1 μl of One Step RT-PCR enzyme mix. Primers and probes specific for the following 17 respiratory viruses were used: AdV [27], PIV 1 to 4 [28], RSV [29], human metapneumovirus (hMPV) [30], pandemic influenza virus A(H1N1)pdm09, seasonal influenza virus A (H1N1, H3N2) (SIA) [31], seasonal influenza virus B (SIB) [32], human coronavirus 229E (HCoV-229E), human coronavirus OC43 (HCoV-OC43), human coronavirus NL63 (HCoV-NL63), human coronavirus HKU1 (HCoV-HKU1) [33], rhinovirus (HRV) [34], human parechovirus (HPeV) [35], and enterovirus (EV) [36]. The 7500 Real Time PCR system from Applied Biosystems was used with the following cycling conditions: 30 min at 50°C for reverse transcription, 15 min at 95°C for denaturation, then 45 cycles for 15 s at 95°C, 30 s at 60°C, and 10 s at 40°C.

### Data analysis

Statview V 5.0 software was used. Pearson’s Chi-square test and Fisher’s exact test were used to compare the prevalence rates of respiratory viruses, along with their geographic distribution and seasonality, and cases of coinfection. We used a two-tailed critical value of alpha = 0.05. A p value below 0.05 was considered to denote statistical significance.

### Ethical considerations

This study was approved by the Gabonese National Ethics Committee (number 0024/CNE/SG/P). Individual oral consent was required for nasal sampling. The test results were transmitted to the participating health centers.

## Results

### Epidemiological findings

1041 specimens were collected from patients with ILI between March 2010 and June 2011. The patients were 515 males (49.5%) and 526 (50.5%) females (sex ratio 0.98; Table 1). Median age was 2 years (range, 10 days to 82 years) and mean age was 6.1 ± 11.6 years. The geographic distribution was as follows: 424 cases (40.8%) in Libreville, 223 (21.4%) in Franceville, 194 (18.6%) in Koulamoutou, and 200 (19.2%) in Oyem. The age distribution was as follows: 810 patients (77.8%) were 0–4 years old, 111 patients (10.7%) 5–15 years old, and 120 patients (11.5%) 16–82 years old (Table 1).

**Table 1:** Demographic characteristics and prevalence of viral infections in ILI patients

**Characteristic**	**Infected**		**Total ILI**	
	**n (%)**	**95% CI**	**N**	**95% CI**
**Sex**				
Male	319 (61.9)	57.7-66.1	515 (49.5)	46.5-52.5
Female	320 (60.8)	56.6-65.0	526 (50.5)	47.5-53.5
**Age group (years)**				
[0–4]	552 (68.1)	64.9-71.3	810 (77.8)	75.3-80.3
[5-15]	46 (41.4)	32.2-50.6	111 (10.7)	8.8-12.6
[16–82]	41 (34.2)	25.7-42.7	120 (11.5)	9.6-13.4
**Towns**				
Libreville	290 (68.4)	64.0-72.8	424 (40.8)	37.8-43.8
Franceville	146 (65.5)	59.3-71.7	223 (21.4)	18.9-23.9
Koulamoutou	82 (42.3)	35.3-49.3	194 (18.6)	16.2-21.0
Oyem	121 (60.5)	53.7-67.3	200 (19.2)	16.8-21.6
**Total**	639 (61.4)	58.4-64.4	1041 (100.0)	

### Virus detection

639 patients (61.4%) were positive for at least one virus (Table 1). The rates of viral positivity were 68.4% in Libreville, 65.5% in Franceville, 42.3% in Koulamoutou and 60.5% in Oyem (Table 1). The rates in Libreville, Franceville and Oyem differed significantly from that in Koulamoutou (*X*^*2*^ = 40.3, p <0.0001).

The rate of viral positivity was similar in males (61.9%) and females (60.8%) (*X*^*2*^ = 0.092, p = 0.76). It was 68.1% in the age group <5 years, compared to 41.4% in the 5- to 15-year age group and 34.2% in the 16- to 82-year age group (*X*^*2*^ = 71.8, p <0.0001) (Table 1).

**Figure 1:**
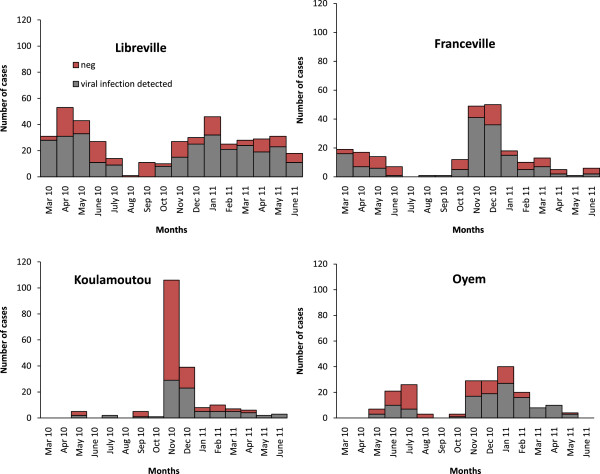
Temporal distribution of ILI by town.

The study began during the long rainy season in March 2010. In Libreville, we observed a significant increase in the rate of virus-positive ILI during the rainy seasons, followed by a decrease in the dry seasons (*X*^*2*^ = 7.8, p = 0.0052) (Figure 1). Seventy per cent of viral infections occurred during the rainy seasons. The only other seasonal difference was that viral infections among children less than 5 years old were more frequent during the rainy season than the dry season, in all four regions (*X*^*2*^ = 6.4, p = 0.0113).

### Virus identification

170 samples contained AdV (16.3%), 155 PIV (14.9%), 128 EV (12.3%), 124 RSV (11.9%), 119 influenza virus (11.4%), 81 HRV (7.8%), 68 HCoVs (6.5%), 19 hMPV (1.8%), and 5 HPeV (0.5%) (Table 2). The most common influenza virus was A(H1N1)pdm09, with 68 cases (57% of all influenza viruses). We detected 51 cases of SIB (43% of all influenza viruses) and no cases of SIA. HCoV-OC43 was the most prevalent HCoV, with 35 cases (51.5% of all HCoVs). PIVs were the second most common viruses, with 155 cases; 33 (21%) were PIV1, 27 (17%) PIV2, 67 (43%) PIV3, and 28 (18%) PIV4. Significant differences in the age distribution were observed for the following viruses: Adv, HCoV-NL63, RSV, EV and HRV (Table 2).

**Table 2:** Prevalence of viral infections by age group

**Viral infection**	**Age group (years)**			**p value**	**Total (N = 1041) n (%)**	**95% CI**
	**[0–4] (N = 810) n (%)**	**[5-15] (N = 111) n (%)**	**[16–82] (N = 120) n (%)**			
AdV	142(17.5)	19(17.1)	9(7.5)	0.02	170(16.3)	14.1-18.5
EV	119(14.7)	3(2.7)	6(5.0)	<0.0001	128(12.3)	10.3-14.3
RSV	109(13.5)	5(4.5)	10(8.3)	0.01	124(11.9)	9.9-13.9
HRV	77(9.5)	3(2.7)	1(0.8)	0.00	81(7.8)	6.2-9.4
A(H1N1)pdm09	55(6.8)	7(6.3)	6(5.0)	0.75	68(6.5)	5.0-8.0
PIV3	58(7.2)	4(3.6)	5(4.2)	0.20	67(6.4)	4.9-7.9
SIB	41(5.1)	7(6.3)	3(2.5)	0.36	51(4.9)	3.6-6.2
HCoV-OC43	33(4.1)	1(0.9)	1(0.8)	0.05	35(3.4)	2.3-4.5
PIV1	29(3.6)	0(0.0)	4(3.3)	0.12	33(3.2)	2.1-4.2
PIV4	26(3.2)	2(1.8)	0(0.0)	0.10	28(2.7)	1.7-3.7
PIV2	23(2.8)	1(0.9)	3(2.5)	0.48	27(2.6)	1.6-3.6
hMPV	15(1.8)	2(1.8)	2(1.7)	0.98	19(1.8)	1.0-2.6
HCoV-NL63	12(1.5)	0(0.0)	5(4.2)	0.03	17(1.6)	0.8-2.4
HCoV-HKU1	10(1.2)	0(0.0)	0(0.0)	0.23	10(0.9)	0.4-1.5
HCoV-229E	6(0.7)	0(0.0)	0(0.0)	0.42	6(0.6)	0.1-1.1
HPeV	5(0.6)	0(0.0)	0(0.0)	0.48	5(0.5)	0.1-0.9
SIA	0(0.0)	0(0.0)	0(0.0)	-	0(0.0)	-

AdV: adenovirus, EV: enterovirus, RSV: respiratory syncytial virus, HRV: human rhinovirus, A(H1N1)pdm09: pandemic influenza virus A, SIA: seasonal influenza virus A, SIB: seasonal influenza virus B, PIV1-4: parainfluenza virus 1–4, HCoV: human coronavirus, hMPV: human metapneumovirus, HPeV : human parechovirus.

As most cases of ILI involved children less than 5 years old (77.8%), and as the viral prevalence was also highest in this age group (68.1%), we focused the remainder of our study on children under 5 years of age. The most common viruses in this age group were Adv (17.5%), PIVs (16.8%), EV (14.7%), RSV (13.5%), and influenza virus (11.9%). PIV3 accounted for 43% of PIVs (Table 3).

### Geographic distribution of respiratory viruses in children less than five years old

The prevalence of the following viruses differed significantly across the four geographic regions: HCoV-OC43, RSV, PIV2, PIV3, HRV, EV and SIB (Table 3). HCoV-OC43 was more prevalent in Libreville and Franceville than in Koulamoutou and Oyem (p = 0.02). PIV2 (p = 0.01) and PIV3 (p = 0.01) were detected significantly more frequently in Libreville than in the other towns. EV (p = 0.01) and HRV (p = 0.00) predominated in the capital and north of Gabon, whereas RSV (p < 0.0001) and SIB (p < 0.0001) were mainly detected in the south (Franceville and Koulamoutou) (Table 3).

**Table 3:** Viral prevalence in children under 5 years old, by geographic area

**Viral infection**	**Towns**				**p value**	**Total (N = 810) n (%)**	**95% CI**
	**Libreville (N = 391) n (%)**	**Franceville (N = 159) n (%)**	**Koulamoutou (N = 98) n (%)**	**Oyem (N = 162) n (%)**			
AdV	63(16.1)	35(22.0)	15(15.3)	29(17.9)	0.37	142(17.5)	14.9-20.1
EV	71(18.2)	16(10.1)	7(7.1)	25(15.4)	0.01	119(14.7)	12.3-17.1
RSV	34(8.7)	36(22.6)	18(18.4)	21(13.0)	<0.0001	109(13.5)	11.1-15.9
HRV	46(11.8)	2(1.3)	6(6.1)	23(14.2)	0.00	77(9.5)	7.5-11.5
PIV3	39(10.0)	9(5.7)	1(1.0)	9(5.6)	0.01	58(7.2)	5.4-9.0
A(H1N1)pdm09	24(6.1)	6(3.8)	9(9.2)	16(9.9)	0.11	55(6.8)	5.1-8.5
SIB	6(1.5)	32(20.1)	1(1.0)	2(1.2)	<0.0001	41(5.1)	3.6-6.6
HCoV-OC43	23(5.9)	7(4.4)	0(0.0)	3(1.8)	0.02	33(4.1)	2.7-5.5
PIV1	20(5.1)	3(1.9)	1(1.0)	5(3.1)	0.11	29(3.6)	2.3-4.9
PIV4	17(4.3)	5(3.1)	3(3.1)	1(0.6)	0.16	26(3.2)	2.0-4.4
PIV2	19(4.9)	3(1.9)	0(0.0)	1(0.6)	0.01	23(2.8)	1.7-3.9
hMPV	9(2.3)	3(1.9)	2(2.0)	1(0.6)	0.61	15(1.8)	0.9-2.7
HCoV-NL63	7(1.8)	3(1.9)	1(1.0)	1(0.6)	0.70	12(1.5)	0.7-2.3
HCoV-HKU1	5(1.3)	1(0.6)	0(0.0)	4(2.5)	0.29	10(1.2)	0.5-1.9
HCoV-229E	0(0.0)	3(1.9)	1(1.0)	2(1.2)	0.09	6(0.7)	0.1-1.3
HPeV	1(0.3)	1(0.6)	0(0.0)	3(1.8)	0.14	5(0.6)	0.1-1.1
SIA	0(0.0)	0(0.0)	0(0.0)	0(0.0)	-	0(0.0)	-

AdV: adenovirus, EV: enterovirus, RSV: respiratory syncytial virus, HRV: human rhinovirus, A (H1N1) pdm09: pandemic influenza virus A, SIA : seasonal influenza virus A, SIB : seasonal influenza virus B, PIV1-4: parainfluenza virus 1–4, HCoV : human coronavirus, hMPV : human metapneumovirus, HPeV: human parechovirus.

### Seasonal distribution of respiratory viruses in children less than five years old

**Figure 2:**
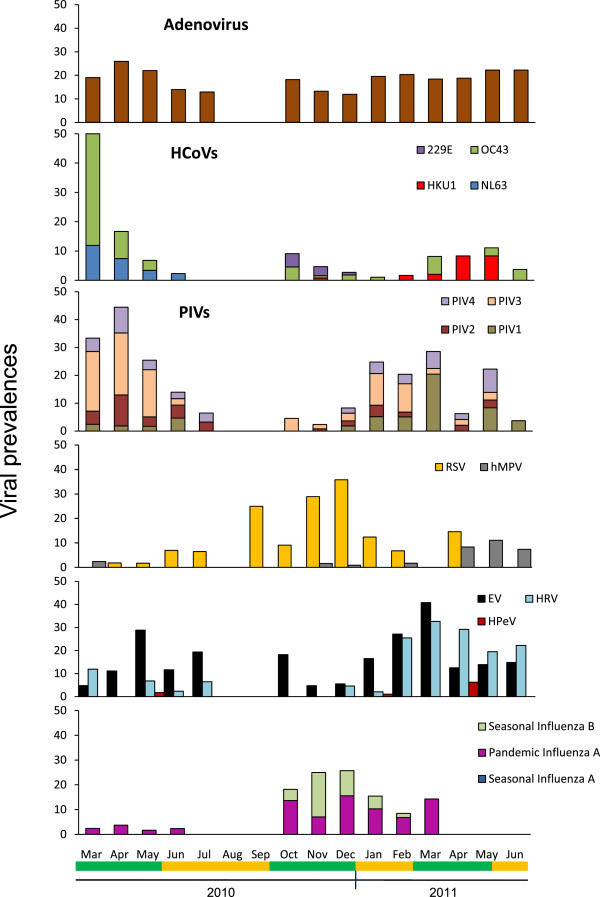
**Viral seasonality in children under five years old.** Short rainy season (short green line), long rainy season (big green line), short dry season (short yellow line), and long dry season (big yellow line).

AdV was detected at a stable rate throughout the study (*X*^*2*^ = 0.006, p = 0.93). Among the HCoVs, only HCoV-OC43 showed significant variations between the rainy and dry seasons (*X*^*2*^ = 9.7, p = 0.0018). The distribution of HCoV species differed between 2010 and 2011. While HCoV-OC43 circulated in both 2010 and 2011, HCoV-NL63 was only detected from March to June 2010. HCoV-229E and HCoV-HKU1 appeared in the short rainy season between October and December, co-circulating with HCoV-OC43. In 2011, we only detected HCoV-OC43 and HCoV-HKU1 (Figure 2). The prevalence of RSV increased during the rainy seasons and declined in the dry seasons (*X*^*2*^ = 8.0, p = 0.0046). RSV was mainly detected (71.6%) in the short rainy season from October to December 2010. The prevalence of A(H1N1)pdm09 and SIB varied across the seasons (*X*^*2*^ = 19.8, p = 0.0014 and *X*^*2*^ = 60.0, p <0.0001, respectively), being highest during the short rainy season in 2010. The decline in the prevalence of influenza virus and RSV between December 2010 and January 2011, corresponding to the transition between the short rainy season and the short dry season, coincided with an increase in EV, HRV and PIV circulation. EV and HRV were seasonally distributed (*X*^*2*^ = 27.4, p <0.0001 and *X*^*2*^ = 80.1, p <0.0001, respectively). Their prevalence was low in the short rainy season in 2010 and increased during the short dry season in 2011, before peaking in March 2011 during the long rainy season (40.8% and 32.7% respectively) (Figure 2). The prevalence of PIVs (44.4%) was highest in April 2010, declining until the next dry season, remaining low during the 2010 short rainy season, and re-increasing during the 2011 short dry season. The prevalence of PIV2 and PIV4 did not vary significantly (*X*^*2*^ = 20.9, p = 0.1397 and *X*^*2*^ = 18.4, p = 0.2434, respectively), while PIV3 was most prevalent during the long rainy season in 2010 (*X*^*2*^ = 63.9, p <0.0001) and PIV1 during the long rainy season in 2011 (*X*^*2*^ = 54.7, p < 0.0001). Fifty-three per cent of hMPV infections were diagnosed during the long rainy season in 2011. We observed only sporadic cases of HPeV infection.

### Co-infections in children less than five years old

Among the 552 samples positive for at least one virus, 174 (31.5%) also contained other viruses (Table 4). We observed 141 (25.5%) cases of dual infection, 32 (5.8%) cases of triple infection, and one case (0.2%) of quadruple infection. The rate of co-infection was similar in males and females (*X*^2^ = 0.0, p > 0.9999). The ratio of mono-infection to multiple infection did not differ across the four sites (*X*^2^ = 1.2, p = 0.76). The patient with quadruple infection (Adv, HCoV-NL63, PIV2 and PIV3) was a 3-year-old child diagnosed in Franceville.

**Table 4:** Prevalence of viral co-infections among children under 5 years old

**Viral infection**	**Single infections (N = 378) n(%)**	**Coinfections (N = 174) n(%)**	**p value**	**Total (N = 552) n(%)**	**95% CI**
AdV	70(18.6)	72(41.4)	<0.0001	142(25.7)	22.1-29.3
EV	44(11.6)	75(43.1)	<0.0001	119(21.6)	18.2-25.0
RSV	77(20.4)	32(18.4)	0.66	109(19.7)	16.4-23.0
HRV	32(8.5)	45(25.9)	<0.0001	77(13.9)	11.0-16.8
A(H1N1)pdm09	37(9.8)	18(10.3)	0.96	55(10.0)	7.5-12.5
PIV3	27(7.1)	31(17.8)	0.00	58(10.5)	7.9-13.1
SIB	25(6.6)	16(9.2)	0.36	41(7.4)	5.2-9.6
HCoV-OC43	18(4.8)	15(8.6)	0.11	33(6.0)	4.0-8.0
PIV1	9(2.4)	20(11.5)	<0.0001	29(5.3)	3.4-7.2
PIV4	11(2.9)	15(8.6)	0.01	26(4.7)	2.9-6.5
PIV2	7(1.8)	16(9.2)	0.00	23(4.2)	2.5-5.9
hMPV	11(2.9)	4(2.3)	0.89	15(2.7)	1.3-4.1
HCoV-NL63	6(1.6)	6(3.4)	0.28	12(2.2)	1.0-3.4
HCoV-HKU1	2(0.5)	8(4.6)	0.00	10(1.8)	0.7-2.9
HCoV-229E	2(0.5)	4(2.3)	0.15	6(1.1)	0.2-2.0
HPeV	0(0.0)	5(2.9)	0.00	5(0.9)	0.1-1.7
SIA	0(0.0)	0(0.0)	-	0(0.0)	-

AdV: adenovirus, EV: enterovirus, RSV: respiratory syncytial virus, HRV: human rhinovirus, A(H1N1) pdm09: pandemic influenza virus A, SIA : seasonal influenza virus A, SIB: seasonal influenza virus B, PIV1-4: parainfluenza virus 1–4, HCoV: human coronavirus, hMPV: human metapneumovirus, HPeV: human parechovirus.

The most common viruses involved in co-infection were PIVs (47.1%), AdV (41.4%), and EV (43.1%), while the main viruses involved in monoinfection were RSV (20.4%) and Adv (18.6%) (Table 4). The most common dual co-infections were EV/HRV 28/174 (16.1%) and AdV/EV 25/174 (14.4%) (Table 5). Influenza virus A(H1N1)pdm09 and SIB were involved in respectively 18 and 16 cases of co-infection (Table 4). RSV was the virus most frequently associated with A(H1N1)pdm09 infection.

**Table 5:** Virus involved in co-infections

**Double infection (total 141)**	**AdV**	**EV**	**RSV**	**HRV**	**p(H1N1)**	**PIV3**	**SIB**	**OC43**	**PIV1**	**PIV4**	**PIV2**	**hMPV**	**NL63**	**HKU1**	**229E**	**HP**
**Adv**																
**EV**	**17**															
**RSV**	**6**	**4**														
**HRV**	**6**	**21**														
**p(H1N1)**	**3**	**4**	**7**													
**PIV3**	**5**	**3**	**1**	**4**	**1**											
**SIB**	**2**	**1**	**4**													
**OC43**	**2**			**1**	**1**	**3**	**1**									
**PIV1**	**1**	**4**		**1**		**1**										
**PIV4**	**6**	**1**		**2**	**1**											
**PIV2**	**2**						**2**	**1**	**7**							
**hMPV**	**1**	**1**		**1**												
**NL63**	**1**															
**HKU1**	**1**	**1**	**1**				**1**									
**229E**			**1**		**1**		**2**									
**HP**		**1**	**1**	**1**												
**AdV/EV/other virus**	8/32*															
**EV/HRV/other virus**	7/32*															
**AdV/NL63/PIV2/PIV3**	1/1**															

AdV: adenovirus, EV: enterovirus, RSV: respiratory syncytial virus, HRV: human rhinovirus, p(H1N1): A(H1N1)pdm09 pandemic influenza virus A, SIB: seasonal influenza virus B, PIV1-4: parainfluenza virus 1–4, OC43: human coronavirus OC43, NL63: human coronavirus NL63, HKU1: human coronavirus HKU1, 229E: human coronavirus 229E, hMPV: human metapneumovirus, HP: human parechovirus, *triple infection, **quadruple infection.

## Discussion

Respiratory viruses causing influenza-like illness are a major source of morbidity and mortality, especially among children [1,2]. In this large nationwide study of ILI in Gabon, central Africa, we found that 61.4% of nasal swabs contained at least one virus. Most previous studies have shown a viral prevalence below 50% [13,37-39], although figures up to 75% have been reported [14,16,17]. Children less than 5 years old accounted for most cases of ILI in our study (77.8%), and 68.1% of them had a documented viral infection (Tables 1 and 2). According to a WHO communication in 2012, 1.2 million children under five years old die each year from pneumonia [40]. It should be noted that Gabonese adults rarely consult for ILI, possibly explaining why they only represented 11.5% of our study population. All these reasons led us to focus on children under five years old.

The viruses most frequently detected in children less than five years old were AdV (17.5%), PIVs (16.8%), EV (14.7%), RSV (13.5%) and influenza virus (11.9%) (Table 3). In previous studies of this age group, the most common viruses were RSV, influenza virus, PIV, EV and HRV [41-43]. The predominant viruses in our study are broadly the same as those diagnosed in neighboring countries, especially among young children. Indeed, in Cameroon, 28.2% of specimens was influenza virus, however, the most common viruses found in children under 5 years of age were RSV (83.9% of specimens) PIV (76.9%) and HRV (64.6%). In the Central African Republic, respiratory viruses were detected in 14.9% of children aged 0–15 years with ILI or acute respiratory illness, including influenza virus in 8.8%, PIV1/3 in 3.3% and RSV 2.7% [44]. Influenza virus was found in 15% of ILI patients in DRC from 2009 to 2011 [45]. Despite the prevalence sometimes different depending on the viruses diagnosed in central Africa countries, the main viruses responsible for ILI were the same. An exception is made for Adv which are predominant in our study. In other tropical country as Brazil, ILI sentinel surveillance from 2000 to 2010 showed that the viral etiology of ILI was respiratory syncytial virus (RSV) in 31% of cases, influenza A in 26%, adenovirus (AdV) in 12%, parainfluenza 2 (PIV-2) in 9%, parainfluenza 3 (PIV-3) in 9%, and influenza B in 9% [9]. In others countries African countries as Kenya, Ghana, in India, or Europe, the prevalence of RSV in children under 5 years of age with ILI ranges between 12% and 60% [13,42,46-48], while that of PIV and adenovirus ranges between 3-22% and 5-25% respectively [13,15,42,46-50]. In Europe, Rhinovirus (HRV) is found in 8% to 12% of young children with ILI [18,37,51].

The prevalence of AdV was far higher here than in many previous studies, in which AdV infection was infrequent [41,51,52]. A similar prevalence of AdV was observed in a Kenyan study in which the median age (1 year) was similar to that of our population (2 years) [13]. In young children, asymptomatic porting has been shown for some respiratory viruses such as adenovirus or rhinovirus [53]. More specifically, asymptomatic adenovirus infections are common in young children [53,54]. In our study, adenoviruses have a predominant prevalence. They are also common in co-infection. Asymptomatic carriage of the virus among young people and their high prevalence in co-infection suggested that they might not be the cause of the disease. RSV, the main etiologic agent of bronchiolitis and pneumonia in young children, has a reported prevalence of 14% to 34% in children with respiratory tract infection, and is particularly frequent in infants less than one year old [42,52,55]. The prevalence of RSV was lower in our study than in many previous reports, in which it reached 50% among children aged between 0 and 4 years [10,56]. However, the children in these latter studies were hospitalized with acute lower respiratory tract infections, whereas the children in our study did not always require hospitalization. RSV was none the less one of the five main viruses detected in our study (13.5%). All PIV types were detected in our study, with a predominance of PIV3 as in several previous studies [57-59]. The prevalence of influenza virus (11.9%) was similar to that observed in other countries (10% to 13%) after the pH1N1 pandemic [17]. Among the influenza viruses, A(H1N1)pdm09 and type B represented 57.3% and 42.7% of cases, respectively. These results could be explained by the fact that our study took place after the global emergence of A(H1N1)pdm09: pandemic influenza and influenza B was more frequent than non A(H1N1)pdm09 influenza A. From November 2010 to February 2011, the subtypes of influenza virus which were mostly prevalent in North Africa, the Near East, Europe and Asia, were the subtype B and the A(H1N1)pdm09 [60]. Our results match those described in the world for this time of year and show that these two types of influenza are predominant.

Some viruses are predominant in urban areas, others in rural areas. Some studies about epidemiology of RSV in Kenya show that the all-cause community-based incidence of SARI among infants is higher in the rural study site, compared to the urban study site [61,62]. In Gabon, Franceville which is a semi rural area has a lower density of population than Libreville and the fact that there is only one regional hospital where patients go, explain why viruses associated with infections in very young children such as the SIB or RSV are more important in this region where the spread would be faster.

ILI is most frequent in the winter months in temperate countries and in the rainy season in tropical countries [11,63]. In Gabon, we found that, among children less than five years old, the number of cases of ILI and the virus detection rate were highest during the rainy seasons from March to May and October to December. The prevalence of PIV infection was highest in April 2010, during the rainy season (Figure 2). Influenza virus and RSV detection increased from November 2010 to December 2010 and then declined in January and February during the short dry season. Our results are compatible with those of a Malaysian study showing seasonal increases in RSV (September to December), PIV1 and PIV3 (March to May), and influenza B (November). Moreover, this latter study showed a correlation between the prevalence of RSV and the number of rainy days [41]. RSV infection has been found to peak in the rainy season in tropical countries such as Ghana, Kenya, Gambia and Colombia [12,42]. Influenza viruses are most prevalent during cold winter months in the temperate northern hemisphere, and during the rainy season in tropical countries such as Senegal, India and Brazil [11,12,20]. EV was detected throughout the year in our study but peaked in May 2010 and March 2011 (rainy seasons). EV seasonality was observed in a study of Caribbean asthmatic children, in which the prevalence increased during the rainy season [64]. In France, EV respiratory infections peaked in children in June-July over several years [65]. In our study, HRV was detected throughout most of the year, as in Madagascar and the West Indies, but peaked from February to April 2011, revealing a seasonal pattern [16,64]. Another study showed that HRV infection in South African young children peaked from February to April, corresponding to autumn months [66]. We detected the highest prevalence of HCoVs (50%) during April 2010 (rainy season). HCoV-OC43 co-circulated with HCoV-NL63 during the long rainy season in 2010, with HCoV-229E during the short rainy season in 2010, and with HCoV-HKU1 during the long rainy season in 2011. A seasonal pattern of HCoVs was also observed in Hong Kong, where HCoV-NL63 infection peaked in September-October, while HCoV-OC43 peaked in December-January [67].

174 (31.5%) of our 552 positive samples contained at least two viruses. Several studies showed coinfections were associated with an increase in disease severity, but the majority of studies didn’t show clinical differences between single and coinfections [68,69]. Thus we compared the clinical data of the patients in single infections with co-infections (data not shown). Our findings do not allow us to conclude that there was a difference in clinical severity between patients with single and multiple infections.

## Conclusions

This study provides unique data on the circulation of viruses associated with upper respiratory tract infections in Gabon. The highest prevalence of viral infections concerned AdV, EV, RSV, HRV, PIV3, SIB. Theses pathogens have the highest prevalence among children under five years old. The responsible viruses were similar to those reported on other continents except for adenoviruses whose prevalence is higher. However adenoviruses can be detected in asymptomatic persons. The prevalence of RSV, influenza virus, PIV3, EV and HRV increased during the rainy season. Ongoing surveillance is necessary to identify circulating strains of influenza virus and thus to respond rapidly to outbreaks. Our findings should help to improve the therapeutic management of patients presenting with influenza-like illness, especially regarding the use of antibacterial agents.

## Competing interests

The authors declare that they have no competing interests.

## Authors’ contributions

Conceived and designed the surveillance: EML, JFD. Performed the laboratory tests: SEL. Analyzed and interpreted the data: SEL, EBN, EML. Established the surveillance programmed: DN. Established the diagnostic testing: CD, JFD. Wrote the manuscript: SEL, EML. All authors read and approved the final manuscript.

## Pre-publication history

The pre-publication history for this paper can be accessed here:

http://www.biomedcentral.com/1471-2334/14/373/prepub
